# Effects of Cognitive Reserve on Cognitive Performance in a Follow-Up Study in Older Adults With Subjective Cognitive Complaints. The Role of Working Memory

**DOI:** 10.3389/fnagi.2018.00189

**Published:** 2018-06-26

**Authors:** Cristina Lojo-Seoane, David Facal, Joan Guàrdia-Olmos, Arturo X. Pereiro, Onésimo Juncos-Rabadán

**Affiliations:** ^1^Department of Developmental and Educational Psychology, Universidade de Santiago de Compostela, Santiago de Compostela, Spain; ^2^Department of Social Psychology and Quantitative Psychology, University of Barcelona, Barcelona, Spain

**Keywords:** structural equation model, education, cognition, lifestyle, working memory, aging, episodic memory

## Abstract

**Objective:** Analyze the effects of CR on cognitive performance in adults with subjective cognitive complaints at follow-up.

**Method:** We analyzed the factorial structure of the three constructs defined in cognitive performance (Episodic memory, Working memory, and General cognitive performance) separately to search for evidence of the invariance of the measurement model. We then developed four structural nested models to analyze the relationship between CR and cognitive performance, measured at baseline and after approximately 18 months, in 266 participants older than 50 years with subjective cognitive complaints.

**Results:** The nested models revealed the following main results: direct effects of CR on all cognitive constructs at baseline and also indirect effects on the same constructs at follow-up, and indirect effects of CR on other cognitive constructs at follow-up via working memory at follow-up.

**Conclusion:** The findings show that the proposed model is useful for measuring the influence of CR on cognitive performance in follow-up studies and that CR has a positive influence on cognitive performance at follow-up via working memory. CR may enhance mechanisms of information processing, favoring performance of tasks involving other cognitive constructs in older adults with subjective cognitive complaints.

## Introduction

Cognitive reserve (CR) refers to how flexibly and efficiently one can make use of the available brain reserve estimated by brain size or neuronal count (Stern, 2002). CR has been defined as a hypothetical construct that can be studied by latent variables related to life experience (Jones et al., 2011). Standard proxies for CR include years of schooling, job complexity or occupational attainment, crystallized intelligence (vocabulary level), literacy, engagement in leisure activities, social and cultural participation and integrity of social networks (Sánchez et al., 2011; Tucker and Stern, 2011; Lojo-Seoane et al., 2012; Giogkaraki et al., 2013). Many of these proxies have been included in a comprehensive questionnaire that quantifies CR by considering three main areas: education, working activity and leisure activities (Nucci et al., 2012). Increased frequency of such activities at any time of life may provide the brain with more resources to enable it to compensate for the damage or stressful situations (Freret et al., 2015; Lenehan et al., 2015b; Gelfo et al., 2017). The CR construct is therefore used to explain why two people can have different clinical manifestations of the same disease (Stern, 2012). The protective effect of CR has been studied in elderly populations such as healthy people, people with subjective memory complaints, and patients with mild cognitive impairment (MCI) (Constantinidou et al., 2014; Mondini et al., 2016; Franzmeier et al., 2017a,b), and some studies have found that CR influences progress to dementia (Ghisletta et al., 2006; Sumowski et al., 2014; Vaughan et al., 2014).

Cross-sectional studies have shown that CR is associated with a high level of cognitive performance, suggesting that individuals with greater CR may have more resources available to confront cognitive decline. Mitchell et al. (2012) found that in healthy and memory-impaired older adults, CR was a valid construct comprising years of education and reading ability and was positively correlated with performance in several cognitive domains such as memory/language, attention and processing speed/executive function. Giogkaraki et al. (2013) used structural equation modelling (SEM) to examine how CR was related to age and cognitive functions in healthy older adults and found that CR exerted a moderating role in decreasing the direct negative effect of age on executive functions and episodic memory.

Subjective cognitive complaints (SCCs) are considered important for establishing subjective markers of decline and for determining the influence of SCCs on changes in cognitive performance. The report of SCCs in adults has been suggested to be an early sign of clinical relevance to determine early symptoms of cognitive impairment (Ávila-Villanueva et al., 2016). SCCs constitute an important criterion for diagnosis of MCI (Petersen et al., 1999; Petersen, 2004; Albert et al., 2011) and individuals with SCCs are more likely to develop dementia than those without (Reisberg and Gauthier, 2008; Mitchell et al., 2014). Taking into account the protective effect of CR, study of the influence over time of CR on cognitive performance of people with SCCs is very important (Van Oijen et al., 2007).

In a cross-sectional study, Lojo-Seoane et al. (2014a) used SEM to examine the relationship between CR as a construct comprising educational and lifestyle variables and cognitive performance in older adults with SCCs. Their findings revealed a model of the relationships between CR and cognitive performance with significant direct effects of CR on working memory (WM), general cognitive performance (GCP) and episodic memory (EM), and a significant indirect effect of CR on episodic memory (EM) through WM, suggesting the importance of this particular construct.

The level of performance of WM tasks has been demonstrated to be a sensitive measure for differentiating between normal cognitive aging and MCI, and between MCI and Alzheimer’s disease (AD) (Economou et al., 2007; Belleville et al., 2008; Gagnon and Belleville, 2011). Evidence for the mediating effect of WM on the relationship between CR and cognitive performance has been obtained in a study comparing a sample of 70 participants with amnestic MCI and a control group of 139 participants (Constantinidou et al., 2014). These researchers examined the relationship between age and decline in EM by taking into account the mediator effects of WM. The findings confirmed that the effect of age on episodic memory was mediated by working memory. Additionally, the mediating role of working memory was more important in the amnestic MCI group.

The relationship between CR and cognitive performance has also been investigated in longitudinal research conducted to examine whether individuals with greater CR may have more resources available with which to confront cognitive decline over time, as well as to test the potential masking of early symptoms of cognitive impairment (Ghisletta et al., 2006; Sumowski et al., 2014; Vaughan et al., 2014; Lenehan et al., 2015a). Lenehan et al. (2015a) conducted a review of studies aimed at analyzing the relationship between education (as a proxy for CR) and age-related cognitive decline. The reviewed studies provide evidence indicating that people with a higher level of education will continue to perform at a higher level of cognitive functioning, which may delay the onset of impairment in the future. Sumowski et al. (2014) conducted a longitudinal study of participants with multiple sclerosis who were evaluated at baseline and after 4–5 years, observing that CR protected against cognitive decline in GCP and memory. Ghisletta et al. (2006) investigated the relationship between engagement with activities (as a proxy for CR) and cognitive performance and observed that participation in cognitively demanding activities was associated with a more gradual decline in cognitive performance in a sample of very old participants (80–85 years of age). Vaughan et al. (2014) examined associations between cognitive activities (such as reading books, playing games, and using computers) carried out during the 12 months prior to the study and cognitive performance in a sample of 393 elderly women, who were tested three times at intervals of 1 year. Although these authors observed an association between CR and cognitive performance at baseline, they did not observe any effect of cognitive activity on latent change in cognitive performance at follow-up. In short, according to the hypothesis of CR, people with greater CR would tolerate greater pathological burden, which would be compensated because they have more neuronal resources. However, due to this greater pathological burden, it is likely that when they initiate the symptoms of dementia, experience a more rapid progression of the disease than those with lower CR. That is, the CR benefits by delaying the onset of cognitive deficits, but when they finally appear, the progression is faster (Wilson et al., 2010; Lojo-Seoane et al., 2012).

As far as we are aware, no longitudinal studies of the effects of CR on cognitive performance in people with SCCs have been conducted to date. Taking into account the importance of this population in relation to cognitive impairment, and considering the previous cross-sectional findings by Lojo-Seoane et al. (2014a), we examined the statistical effects of CR (measured at baseline) on the natural course of cognitive performance in a sample of older adults with SCCs, over a period of about 18 months. In accordance with Lojo-Seoane et al. (2014a), the CR construct comprised educational and lifestyle variables. The effect of CR was tested in the three constructs of cognitive performance: EM, WM, and GCP. The specific aims of this research were: (A) To determine whether the relationships between CR and cognitive performance (positive direct effects of CR on WM, GCP, and EM and indirect effect of CR on EM through WM) tested in the previous model including baseline data (Lojo-Seoane et al., 2014a) were maintained in the analysis of the corresponding follow-up data. (B) To analyze the role of CR on cognitive performance at follow-up through the three cognitive domains measured at baseline. (C) To test the role of working memory at follow-up to confirm it mediating role. In keeping with previous research, we predicted that at follow-up (1) the CR model proposed in the previous study will be useful for measuring the relationships between CR and cognitive performance at follow-up, (2) CR will have significant and positive effects on cognitive performance at follow-up, and (3) CR will help to maintain a higher level of WM, which will have a positive influence on GCP and episodic memory over time.

## Methodology

### Participants

The study included 266 participants with SCCs, 180 women (67.67%) and 86 men (32.33%), who completed the baseline and one follow-up assessment within the ongoing longitudinal Compostela study carried out in public primary health care centers in Santiago de Compostela (Spain) (Juncos-Rabadán et al., 2012). However, 100 participants assessed at baseline did not participate in the follow-up assessment. The main reasons for attrition were lack of motivation (47%), mobility difficulties (32%), morbidity (10%), possible dementia (5%), health (4%), and mortality (2%) (Facal et al., 2016). All participants were referred by general practitioners according to criteria of subjective cognitive complaints of the study (participants spontaneously reported that their memory was not as good as before). The educational levels of the participants ranged from basic schooling (0–4 years of education) to university studies (+13 years of education) (mean 9.63 ± 4.45). In addition, the participants did not fulfill any of the following exclusion criteria at baseline: prior diagnosis of depression or other psychiatric disturbances (according to DSM-IV criteria), according to the medical records provided by general practitioners; prior diagnosis of neurological disease, including probable AD or other types of dementia (according to NINCDS-ADRDA, Dubois et al., 2007; and DSM-IV criteria, American Psychiatric Association [APA], 1994); previous brain damage or brain surgery; undergoing chemotherapy; prior diagnosis of diabetes type II; sensorial or motor disturbances; or consumption of substances previous that might affect normal performance of the tasks. Descriptive statistics for the observed distribution of demographic variables and continuous proxies for CR and frequency of categorical proxies for CR are shown in **Table 1**.

**Table 1:** Descriptive statistics for the observed distribution of demographic variables and continuous proxies for CR and frequency of categorical proxies for CR (WAIS III = Wechsler Adult Intelligence scale) (*n* = 266).

	Mean	Standard deviation	Range
Time of Follow-up (months)	18.67	2.73	15–25
Age (years)	66.68	9.05	50–87
Years of education	9.63	4.45	1–22
WAIS III vocabulary test	48.53	13.71	15–75
Peabody picture-vocabulary test	61.63	17.41	7–94
**Categorical variables**			
	**Category**	**Frequency**	
Occupational attainment	No occupation	0.4	
	Unqualified worker	40.6	
	Housewife	20.7	
	Qualified worker	29.7	
	Other	8.6	
Reading habits	Never	15.0	
	Occasionally	12.4	
	Once or twice a week	18.8	
	Everyday	53.8	
Social activities	Never	30.5	
	Occasionally	28.6	
	Often	22.2	
	Always	18.8	
Cultural activities	Never	66.5	
	Occasionally	9.8	
	Often	10.5	
	Always	13.2	

### Instruments

An *ad hoc* questionnaire was constructed and administered to the study participants in person. The following measures were considered observable indicators of CR: (a) total number of years of formal education; (b) occupational attainment, which evaluates the complexity of the profession to which the participants have dedicated most of their working life, according to the protocol outlined in a project entitled “Network for efficiency and standardization of dementia diagnosis” (NEST-DD) (Garibotto et al., 2008) on a scale of 1 to 6 (where 1 = no occupation, 2 = unqualified worker, 3 = housewife, 4 = qualified worker, shop-keeper, low-ranking civil servant, employee, small business employee, office worker or sales person, 5 = middle-ranking civil servant or manager, small business owner, teacher or specialist in subordinate position, and 6 = high-ranking civil servant or director, university lecturer, self-employed with high level of responsibility); (c) reading habits, a measure that evaluates the frequency of reading during the last 3 years via one question with responses on a scale of 1–4, where 1 = never (less than once a month), 2 = occasionally (once or twice a month), 3 = often (once a week), and 4 = frequently (every day); (d) frequency of social and cultural activities, which evaluates participation in these types of activity during the last 3 years via two questions with responses on a scale of 1 to 4 [where 1 = never (less than once a month), 2 = occasionally (once or twice a month), 3 = often (once a week), and 4 = frequently (every day)]; (e) level of vocabulary, as index of crystallized intelligence, evaluated by two measures: the vocabulary test of the Wechsler Adult Intelligence Scale (WAIS III) (Wechsler, 2001), which has a test–retest reliability of between 0.60 and 0.80 and a concurrent validity score of 0.82 with the Stanford–Binet test, and the Peabody picture vocabulary test (Dunn and Dunn, 1981), which has a test–retest reliability of 0.77 and a concurrent validity of 0.86 with the Van Alstyne Picture Vocabulary Test and Full- Range Picture Vocabulary Test Quick Test, and of 0.64 with WAIS Vocabulary Subtest.

Episodic memory (EM), a construct including acquisition and recall of verbal material, was evaluated with the Spanish version of the California Verbal Learning Test with norms for age groups (CVLT, Delis et al., 1987, Spanish version by Benedet and Alejandre, 1998). The CVLT consists of a 16-word list (List A) with four words from each of four semantic categories. List A is presented five times in succession, and another 16-word list (List B) is then presented once, as a distractor. The subject is asked to freely recall the words from List A (short term free recall) and is then provided with some semantic categorical cues (short term cued recall). After a 20-min delay, during which non- verbal tests are administered, free and semantically cued recall trials are again administered with List A (free and cued recall in long terms). This is concluded with a recognition trial that requires correct identification of the 16 original words (List A) from among several distractor words, some of which were from List B, as well as other semantically related and unrelated words that were not on either list. This test has proven to have adequate reliability (0.94) and validity (explaining 67% of the variance). The scores for free and cued recall in the short and long term were used as observable measures.

Working memory (WM), i.e., the capacity to maintain and simultaneously manipulate information, was evaluated by two span tasks: (a) the counting span task (Case et al., 1982), and (b) the listening span task (Pickering et al., 1999), which is an adaptation of the reading span task developed by Daneman and Carpenter (1980). WM span tasks are unambiguous measures of WM that necessarily involve retention of information while other information is being processed, fitting well with the definition of WM as the global capability to simultaneously maintain and manipulate information (Belleville et al., 2008; Aretouli and Brandt, 2009). WM span tasks have been used in participants with different levels of cognitive impairment, normal controls and patients with MCI or AD (Gagnon and Belleville, 2011). In the Counting Span task, a random number of target items (dark blue circles) were shown on slides in which non-target items sharing a feature with the targets were presented at the same time (light blue circles and dark blue squares). The participants were asked to count out loud and then recall the number of dark blue circles shown in each slide, in the same order, as soon as a recall cue appeared on the screen (Case et al., 1982). The task consisted of 21 experimenter-paced trials ranging from two to eight figures presented in an ascending format. The test was terminated when the participants failed to remember the number of dark blue circles in three trials with a given number of stimuli. Administration time was about 5 min, depending on the level of impairment. The listening span task is an adapted version of the reading span task (Daneman and Carpenter, 1980), in which participants listen to (rather than read) the stimuli. A group of sentences is read aloud by the experimenter, and the participant must confirm whether each sentence is true or false. Once the group of sentences has been presented, a recall cue appears on the screen and the participant must remember the last word of each sentence in the same order that the sentences were presented. Participants are told to respond “true” or “false” according to the content of each sentence (i.e., “lions have four legs”: true), then to repeat the last word of the sentence (“legs”) and finally to respond “true” or “false” and the last word (“true, legs”). The task consisted of 15 experimenter-paced trials ranging from two to six sentences presented in an ascending format. The number of sentences in each trial increased by one every three trials. The test was terminated when participants failed to remember the final words in three trials for a particular list length. Administration time is about 5 min, depending on the level of impairment. For working memory construct, the observable measures were the total number of correct items and the total number of completed series in each span task.

General cognitive performance (GCP) was evaluated by the following tests: (a) the Mini Mental State Exam (MMSE) (Folstein et al., 1975; Spanish version by Lobo et al., 1999), which has proven to have good sensitivity (89.8%) and specificity (75.1%); and (b) the Spanish version of the Cambridge Cognitive Examination (CAMCOG- R) (Roth et al., 1986; Spanish version by López-Pousa, 2003) with norms for age and educational groups (Pereiro et al., 2015), which has proven to have a reliability of 0.81 and a convergent validity of 0.71. Both the MMSE and the CAMCOG-R include subtests that assess cognitive abilities, such as language comprehension, concentration and numerical calculation, abstract thinking, immediate auditory memory and visuo-motor coordination (praxis), similar to those included in the concept of general intelligence or intelligence quotient (IQ). The total score for the CAMCOG-R ranges from 0 to 105 points distributed among the following domains: orientation (10 points), language (30 points), memory (27 points), attention and calculation (9 points), praxis (12 points), abstraction (8 points) and perception (9 points). The CAMCOG-R includes an additional domain for evaluating executive function. As observable GCP variables, we used the total MMSE scores, the total CAMCOG-R scores and the specific CAMCOG-R scores for orientation and attention, which are particularly sensitive to aging and cognitive impairment (Cullum et al., 2000).

### Procedure

The subjects participated in a follow-up study with two evaluations, one at baseline and other after about 18 months, an interval that is similar to and even slightly longer than that used in the Mayo Clinic Study of Aging (about 15 months) in which cognitive changes were observed at follow-up of a sample of adults aged 70–89 at baseline (Machulda et al., 2013). After the baseline assessment, all participants were informed that they would be called for a follow-up evaluation, and appointments were arranged by telephone. Evaluations were made by trained psychologists in the primary care center and were carried out within the framework of a broader large evaluation protocol. The complete evaluations comprising neuropsychological tests and interviews were conducted in three sessions, each lasting an hour and a half, on three different days, and the instruments used for this study were included in these sessions. The tests were administered in the following order: questionnaire for CR measures, MMSE and CAMCOG for GCP, CVLT for episodic memory, WAIS vocabulary test and working memory tasks. The time in months between the two assessments varied slightly depending on the availability of the participants (mean: 18.67, standard deviation: 2.73). The same neuropsychological assessment and interviews were used at baseline and follow-up evaluation, to enable comparison of the two evaluations.

The study was approved by the Clinical Research Ethics Committee of the Xunta de Galicia (Spain) and was conducted in accordance with the provisions of the Declaration of Helsinki, as revised in Seoul 2008. The participants were informed of the study objectives and procedures involved and were required to sign an approved informed consent before each evaluation.

### Theoretical Structural Model

**FIGURE 1:**
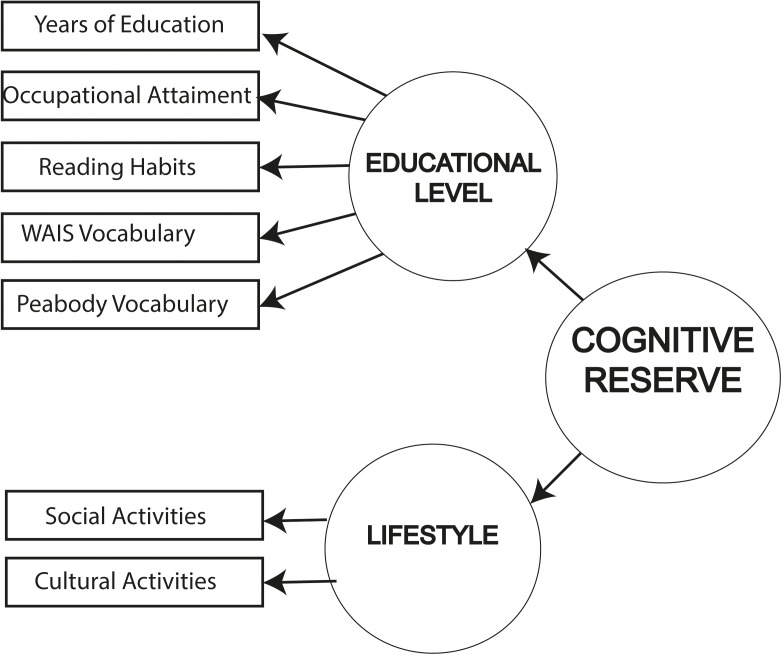
Exogenous measurement model for CR.

**FIGURE 2:**
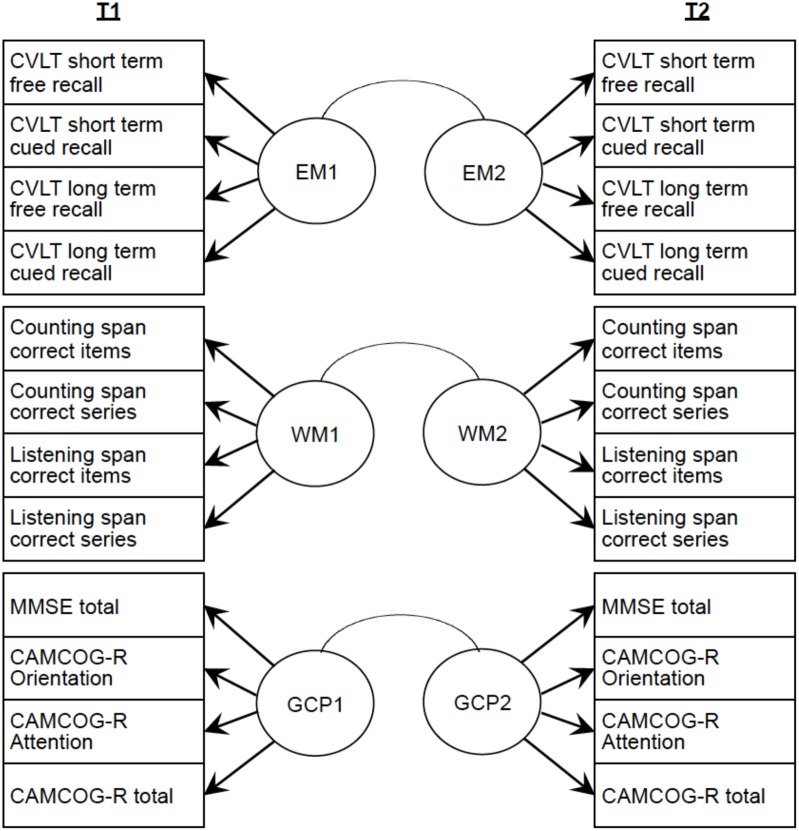
Endogenous model for the three latent variables GCP, EM, and WM.

The proposed structural model is based on the model presented by Lojo-Seoane et al. (2014a). The CR construct was defined from an exogenous measurement model composed of a second-order factor formed by two latent variables: (a) educational level, which includes the observable variables years of education, occupational attainment and reading habits as the main variables related to education (Stern, 2009; Giogkaraki et al., 2013; Lojo-Seoane et al., 2014a), and scores obtained in the WAIS III vocabulary and Peabody tests as main variables related to crystallized intelligence (Tucker and Stern, 2011); and (b) lifestyle, formed by the inter-related variables frequency of social activities and frequency of cultural activities, which have been shown to be the main proxies for engagement in active living (Verghese et al., 2006; Wilson et al., 2007; Stern, 2009; Lojo-Seoane et al., 2014a). The structural model included the direct effect of CR on three cognitive constructs, EM, WM, and GCP, as an endogenous measurement model. This enables reflective analysis of the relationship between the CR construct and these three cognitive performance domains. In order to define these cognitive constructs, we proposed a formative system of indicators for each construct. For EM, the endogenous structure of the complete model included verbal EM measures of short- and long-term free recall and cued recall from the CVLT. WM grouped the scores obtained in two different tasks - counting and listening span – which measure simultaneous storage and processing. GCP included four measures of GCP: the MMSE total score, the CAMCOG-R total score, the Orientation and Attention scores. The exogenous and endogenous models are shown in **Figures 1**, **2**.

**FIGURE 3:**
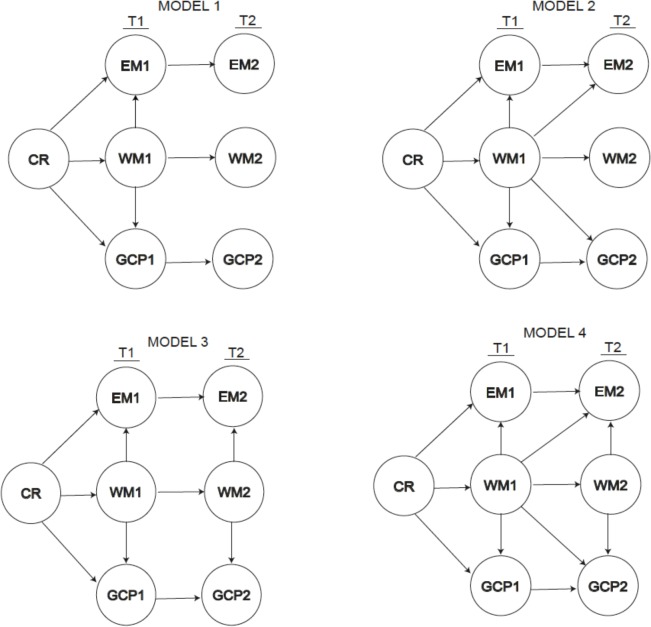
Path diagram for the four models proposed.

Prior to developing the nested structural models with CR, we constructed Model 0, which includes the correlations between different cognitive domains at each moment and which tests the effects of cognitive performance domains at baseline (EM1, WM1, and GCP1) on the same cognitive performance domains at follow-up (EM2, WM2, and GCP2). We can thus explore the magnitude of the cognitive domain correlations without the mediated effect of CR. In order to test the effects of CR on cognitive performance at follow-up, we proposed four nested models (**Figure 3**) representing the latent variables (excluding the measurement models to facilitate the representation) that included different relationships between CR and the cognitive performance constructs (EM, WM, and GCP) at baseline and at follow-up (Benyamini and Roziner, 2008). In the figures showing these models, we represent only the covariance structure to simplify illustration of the structural parameters. As already explained, we used nested models, i.e., we considered Model 1 as the base model and tested other nested models that use the same constructs but specify additional parameters to be estimated. The correlation matrix can be found in the following repository: osf.io/x5uhd.

To test hypotheses 2 and 3, we developed four nested models including the mediated effect of CR. The first model (Model 1) includes the direct effect of CR on the three cognitive constructs at baseline (Time 1, T1): episodic memory (EM1), working memory (WM1) and general cognitive performance (GCP1), and the indirect effects of CR on EM1 and GCP1 via WM1. We added the direct effects of cognitive constructs at baseline (T1) on the same constructs at follow-up (Time 2, T2) to analyze whether cognitive performance in a construct relates to cognitive performance on the same construct and the indirect effect of the CR over time. We constructed Model 2 by adding the direct effect of WM1 on EM2 and GCP2 to Model 1 to test the influence of the processing resources (WM) assessed at baseline on EM2 and GCP2. We constructed Model 3 from Model 1 by incorporating an indirect effect of WM2 on the corresponding EM2 and GCP2 to check the mediating effect of working memory at T2. Finally, we constructed Model 4 from models 1, 2, and 3 by incorporating all of the effects in order to test the influence of working memory at both times.

### Statistical Analysis

We used a general linear model to compare performance on all cognitive variables of participants who attended and those who did not attend the follow-up assessment.

We implemented the SEM by using MPLUS 5.1 (Muthén and Muthén, 1998–2007). To evaluate how the models fit the equations, we used the most commonly accepted parameters (Schreiber et al., 2006), in addition to the specific estimates for each parameter: the CFI (Comparative Fit Index), TLI (Tucker Lewis Index) and the RMSEA (Root Mean Square Error of Approximation). Values of CFI equal to or higher than 0.95 are recommended as indicators of global fit. For the RMSEA, the fit is considered excellent when the value is below 0.06 and is considered adequate when the value is between 0.06 and 0.08; the confidence interval is estimated for better interpretation. We also used the χ^2^ test of goodness of fit to analyze the structural fit between the matrix of initial correlations (*R*) between the observed variables and the reproduced matrix Σ of the same correlation coefficients (from the decomposition rules derived from the structures of the equations that are defined on specifying the model). Although generally known, it must be taken into account that the structural parameters are estimated by minimizing the differences (*R* – Σ). The ratio of χ^2^ to *df* is useful in studies of nested models and an appropriate fit is assumed when the value is less than 2. Finally, we considered the Akaike Information Criterion (AIC) and Bayesian Information Criterion (BIC) values. Low values of these criteria indicate good fits.

## Results

The general linear model comparing cognitive performance of participants who attended follow-up assessment and those who did not attend showed no significant differences in GCP variables {MMSE total = [*F*_(1,364)_ = 3.91, *p* = 0.05, ${\text{η}}_{\text{p}}^{2}$ = 0.011, observed power = 0.505], CAMCOG-R Orientation = [*F*_(1,364)_ = 2.47, *p* = 0.11, ${\text{η}}_{\text{p}}^{2}$ = 0.007, observed power = 0.348], CAMCOG-R Attention = [*F*_(1,364)_ = 0.87, *p* = 0.35, ${\text{η}}_{\text{p}}^{2}$ = 0.002, observed power = 0.154], CAMCOG-R total = [*F*_(1,364)_ = 2.53, *p* = 0.11, ${\text{η}}_{\text{p}}^{2}$ = 0.007, observed power = 0.355]}, or significant differences in WM {Counting span correct items = [*F*_(1,364)_ = 0.27, *p* = 0.6, ${\text{η}}_{\text{p}}^{2}$ = 0.001, observed power = 0.081], Counting span correct series = [*F*_(1,364)_ = 0.8, *p* = 0.37, ${\text{η}}_{\text{p}}^{2}$ = 0.002, observed power = 0.165], Listening span correct items = [*F*_(1,364)_ = 0.96, *p* = 0.32, ${\text{η}}_{\text{p}}^{2}$ = 0.003, observed power = 0.165], Listening span correct series = [*F*_(1,364)_ = 0.20, *p* = 0.64, ${\text{η}}_{\text{p}}^{2}$ = 0.001, observed power = 0.074]}. Significant differences were observed between those who attended the follow-up and those who did not in the EM variables {CVLT short term free recall = [*F*_1,364)_ = 7.69, *p* = 0.006, ${\text{η}}_{\text{p}}^{2}$ = 0.021, observed power = 0.79], CVLT short term cued recall = [*F*_(1,364)_ = 12.08, *p* = 0.001, ${\text{η}}_{\text{p}}^{2}$ = 0.032, observed power = 0.934], CVLT long term fee recall = [*F*_(1,364)_ = 8.27, *p* = 0.004, ${\text{η}}_{\text{p}}^{2}$ = 0.022, observed power = 0.818], CVLT long term cued recall = [*F*_(1,364)_ = 7.91, *p* = 0.005, ${\text{η}}_{\text{p}}^{2}$ = 0.021, observed power = 0.801]}. We also tested whether the two groups differed in two important CR proxies, i.e., years of formal schooling [*F*_(1,364)_ = 0.03, *p* = 0.854] and the WAIS Vocabulary Test [*F*_(1,364)_ = 1.69, *p* = 0.195]. In short, there were no differences in two important CR proxies between participants who attended and those who did not attend the follow-up, and only differences in the measures of episodic memory were observed, although with very a small effect size.

Mean values, standard deviations, skew and kurtosis, differences between baseline and follow-up scores and effect size (standardized measure of Cohen’s *d*) for neuropsychological measures are shown in **Table 2**. Although we observed statistically significant differences in performance on most neuropsychological measures between assessments, the size of the effect was so small that these differences were negligible. Moreover, although the skew and kurtosis parameters for some cognitive variables were outside the normal range, the residual values of all the proposed models were adjusted to normal and we therefore assumed that the data were normally distributed (Searly, 1987).

**Table 2:** Minimum and maximum possible score in the tests, mean values and standard deviations (in parenthesis) for neuropsychological measures and *t*-test comparison between scores measured at baseline (T1) and at follow-up (T2) (*n* = 266).

	Minimum-maximum score	T1	Skew T1	Kurtosis T1	T2	Skew T2	Kurtosis T2	*t* Student	Effect Size
**Episodic memory**									
CVLT short term free recall	0–16	8.94 (3.81)	−0.350 (0.153)	−0.404 (0.306)	9.72 (4.11)	−0.497 (0.150)	−0.315 (0.298)	4.33^*^	0.09
CVLT short term cued recall	0–16	10.21 (3.53)	−0.605 (0.153)	−0.017 (0.306)	10.84 (3.62)	−0.748 (0.150)	0.002 (0.298)	5.12^*^	0.11
CVLT long term free recall	0–16	9.85 (3.92)	−0.578 (0.153)	−0.084 (0.306)	10.34 (4.28)	−0.765 (0.150)	−0.159 (0.298)	4.01^*^	0.09
CVLT long term cued recall	0–16	10.51 (3.56)	−0.659 (0.153)	0.103 (0.306)	11.19 (3.91)	−0.906 (0.150)	0.190 (0.298)	3.99^*^	0.07
**Working memory**									
Counting span correct items	0–60	26.81 (12.04)	−0.504 (0.153)	0.235 (0.306)	30.26 (13.12)	−0.667 (0.153)	0.135 (0.304)	4.78^*^	0.10
Counting span correct series	0–6	2.49 (1.27)	0.532 (0.153)	−0.199 (0.306)	2.73 (1.31)	−0.310 (0.153)	−0.740 (0.304	2.77^**^	0.02
Listening span correct items	0–60	13.69 (7.73)	0.072 (0.153)	−0.990 (0.306)	14.71 (8.94)	−0.115 (0.153)	−1.230 (0.304)	4.23^*^	0.08
Listening span correct series	0–6	1.28 (1.15)	1.543 (0.153)	5.061 (0.306)	1.44 (1.16)	−1.004 (0.153	1.390 (0.304)	2.79^**^	0.03
**General cognitive performance**									
MMSE total	0–30	27.41 (2.23)	−1.162 (0.153)	0.992 (0.306)	27.14 (2.75)	−1.381 (0.149)	1.667 (0.298)	2.89^**^	0.04
CAMCOG-R Orientation	0–10	9.49 (0.78)	−1.588 (0.153)	2.206 (0.306)	9.48 (1.04)	−2.471 (0.149)	5.920 (0.298)	0.12	
CAMCOG-R Attention	0–9	7.17 (1.84)	−0.935 (0.153)	0.256 (0.306)	7.35 (1.85)	−0.756 (0.149)	−0.758 (0.298)	1.93^**^	0.02
CAMCOG-R total	0–105	85.56 (9.27)	−0.530 (0.153)	−0.079 (0.306)	86.97 (10.63)	−0.897 (0.149)	0.483 (0.298)	4.11^*^	0.09

*CVLT, California Verbal Learning Test; MMSE, Mini-Mental State Examination; CAMCOG-R, Cambridge Cognitive Examination-Revised; T1, Baseline; T2, Follow-up.*^∗^*p < 0.01,*^∗∗^*p < 0.05.*

We examined the factorial structure of the three constructs of cognitive performance (EM, WM, and GCP) separately to search for any evidence for the invariance of the measurement model between T1 and T2 for each construct. The SEM fit indices for the three constructs were as follows: for EM, a value of χ^2^ = 321.55 was obtained; *p* = 0.27 (RMSEA = 0.001 [90% CI = 00–003], CFI = 0.99, SMRS = 0.01) and all factor loadings were statistically significant (*p* < 0.001); for WM, χ^2^ = 455.02; *p* = 0.41 (RMSEA = 0.001 [90% CI = 00–003], CFI = 0.99, SMRS = 0.008) and all factor loadings were statistically significant (*p* < 0.001); and for GCP, χ^2^ = 328.12; *p* = 0.27 (RMSEA = 0.006 [90% CI = 00–003], CFI = 1, SMRS = 0.01) and again, all factor loadings were statistically significant (*p* < 0.001). These results show the invariance of the measurement model as defined for each construct and its structural stability in relation to the measures at T1 and T2.

Prior to studying the various models proposed, we examined the factorial structure of the two measurement models (exogenous for CR and endogenous for EM, WM, and GCP) including estimation of the correlation between two times to search for any evidence of stability of the measurement model between T1 and T2 for each construct. The fits for both measurement models were adequate: χ^2^ = 252.87; df = 361; *p* = 0.9912; RMSEA = 0.042; CFI = 0.961; TLI = 0.949, for the exogenous model and χ^2^ = 292.23; df = 276; *p* = 0.240, for the endogenous model. The correlations between T1 and T2 [*r* = 0.734 (*p* < 0.001) for EM, *r* = 0.856 (*p* < 0.001) for WM, and *r* = 0.614 (*p* < 0.001) for GCP] indicate the stability of the measures.

After testing the fit of the measurement models, we analyzed the hypothesized models that included structural effects between exogenous and endogenous factors. For the parameter estimations of all the models, we assumed the correlations between the variances of the measurement residuals of all the observable variables (exogenous and endogenous) as free parameters, in order to improve the fit of the data to the model and the total variance explained. Thus, in addition to the remaining usual assumptions of the SEMs (multivariate normal distribution), the statement *E(*δ_i_δ_j_*)* ≠ *E(€*_i_*€*_j_*)* ≠ *0* holds true for all models.

**FIGURE 4:**
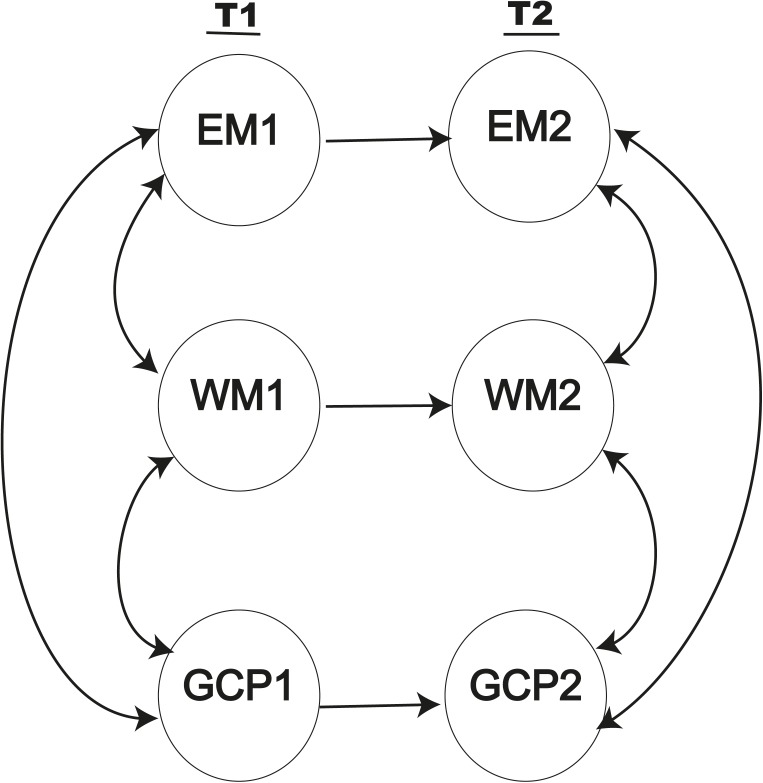
Baseline model including only direct effects between the same measures at the two assessment times.

Model 0 (**Figure 4**) showed significantly positive correlations between the different domains of cognitive performance at both baseline and follow-up. This model also showed significant direct effects of EM1 on EM2 (γ = 0.289; *p* < 0.001), WM1 on WM2 (γ = 0.321; *p* < 0.001) and GCP1 on GCP2 (γ = 0.402; *p* < 0.001). The goodness-of-fit indices for this model were not satisfactory, and the values of the fit indices CFI (0.89), TLI (0.913), and RMSEA (0.13) were also not adequate. Consequently, in addition to the direct effects estimated, we needed to improve model 0 by adding direct and indirect effects between measures, and the exogenous measurement model should also be included to evaluate the impact of CR on the aforementioned measures.

Analysis of the proposed nested models (**Figure 3**) yielded different values for each fit. The results obtained for maximum likelihood estimations with the aforementioned restrictions are summarized in **Table 3**. From the data presented in the table, we can infer that Model 3 provided the best fit. Indeed, both CFI (0.966) and TLI (0.976) values are adequate, as is the RSMEA estimation (0.059). The value of χ^2^ is statistically significant (*p* < 0.001), but the value of χ^2^/degrees of freedom is very favorable (1.94). The AIC and BIC values are the lowest of all the models analyzed, confirming that Model 3 is the best of those evaluated.

**Table 3:** Fitting indices derived from robust ML estimation applied to the structural models.

Indicator	Model 1	Model 2	Model 3	Model 4
**χ^2^**	407.13	365.42	234.74	399.84
**df**	123	121	121	119
			
***P***	<0.001	<0.001	<0.001	<0.001
**Ratio (<2)**	3.31	3.02	1.94	3.36
**CFI (>0.95)**	0.848	0.892	0.966	0.901
**TLI (>0.95)**	0.871	0.913	0.976	0.911
**AIC (<)**	9279,68	9276.70	8280.54	9286.69
**BIC (<)**	9352,11	9287.02	8362.96	9366.29
**RMSEA (<0.06)**	0.093	0.087	0.059	0.082

*The values in parentheses in the first column are values that would indicate excellent fits for each measurement (n = 266).*

Although some models yielded poor fits, we have included the values of the structural parameters for all the models (in **Table 4**) to simplify illustration of the structural effects specified in every model.

**Table 4:** Parameter estimation of the covariance structure of each model.

	Exogenous						
	CR	EM1	WM1	GCP1	EM2	WM2	GCP2
Model 1 All λ_ij_ between 0.341 and 0.623 (*p* < 0.001)							
EM1	0.442		0.033^**^				
WM1	0.312						
GCP1	0.297		0.219				
EM2		0.512					
WM2			0.672				
GCP2				0.601			
Model 2 All λ_ij_ between 0.377 and 0.701 (*p* < 0.001)							
EM1	0.031^**^		0.411				
WM1	0.431						
GCP1	0.328		0.372				
EM2		0.711	0.102^**^				
WM2			0.585				
GCP2			0.301	0.423			
Model 3 All λ_ij_ between 0.309 and 0.687 (*p* < 0.001)							
EM1	0.027^**^		0.171^*^				
WM1	0.244						
GCP1	0.299		0.159^*^				
EM2		0.270				0.192^*^	
WM2			0.251				
GCP2				0.211		0.166^*^	
Model 4 All λ_ij_ between 0.361 and 0.614 (*p* < 0.001)							
EM1	0.034^**^		0.378				
WM1	0.548						
GCP1	0.499		0.428				
EM2		0.602	−0.04^**^			0.231	
WM2			0.626				
GCP2			−0.09^**^	0.577		0.399	
	CR	EM1	WM1	GCP1	EM2	WM2	GCP2

^∗∗^*Not significant,*^∗^*p < 0.05, others p < 0.001;* λ*_ij_ are the factorial coefficients.*

Model 1 reflects significant direct effects of CR on EM1 (γ = 0.442; *p* < 0.001), WM1 (γ = 0.312; *p* < 0.001) and GCP1 (γ = 0.297; *p* < 0.001). CR also had an indirect effect on GCP1 via WM1 (β = 0.219; *p* < 0.001) but not on EM1 (β = 0.033; *p* = 0.512). This model also revealed significant direct effects of cognitive performance constructs at baseline on cognitive performance constructs at follow-up (EM1–EM2, β = 0.512, *p* < 0.001; WM1–WM2, β = 0.672, *p* < 0.001; GCP1–GCP2, β = 0.601, *p* < 0.001).

In Model 2, the significant direct effect of CR on WM1 (γ = 0.431; *p* < 0.001) and GCP1 (γ = 0.328; *p* < 0.001) was maintained, as in Model 1, but the effect on EM1 was not (γ = 0.031, *p* = 0.421). Significant direct effects of WM1 on WM2 (β = 0.585; *p* < 0.001) and also on GCP2 (β = 0.301; *p* < 0.001), but not on EM2 (β = 0.102, *p* = 0.811), were observed. Significant direct effects of the cognitive constructs were maintained at baseline on the corresponding constructs at follow-up, as in Model 1, with similar intensity and equal direction.

Model 3 showed similar effects to those in Model 2 at baseline and for the baseline constructs on those at follow-up. This model also revealed significant indirect effects of WM2 on EM2 and GCP2 (β = 0.192; *p* < 0.05; and β = 0.166; *p* < 0.05, respectively).

Model 4 showed similar effects to those in Models 2 and 3 and also revealed significant indirect effects of WM2 on EM2 and GCP2 (β = 0.231; *p* < 0.001; and β = 0.399; *p* < 0.001, respectively); however, the direct effects of WM1 on EM2 and GCP2 were not significant (β = −0.04, *p* = 0.551; and β = −0.091, *p* = 0.577, respectively).

**FIGURE 5:**
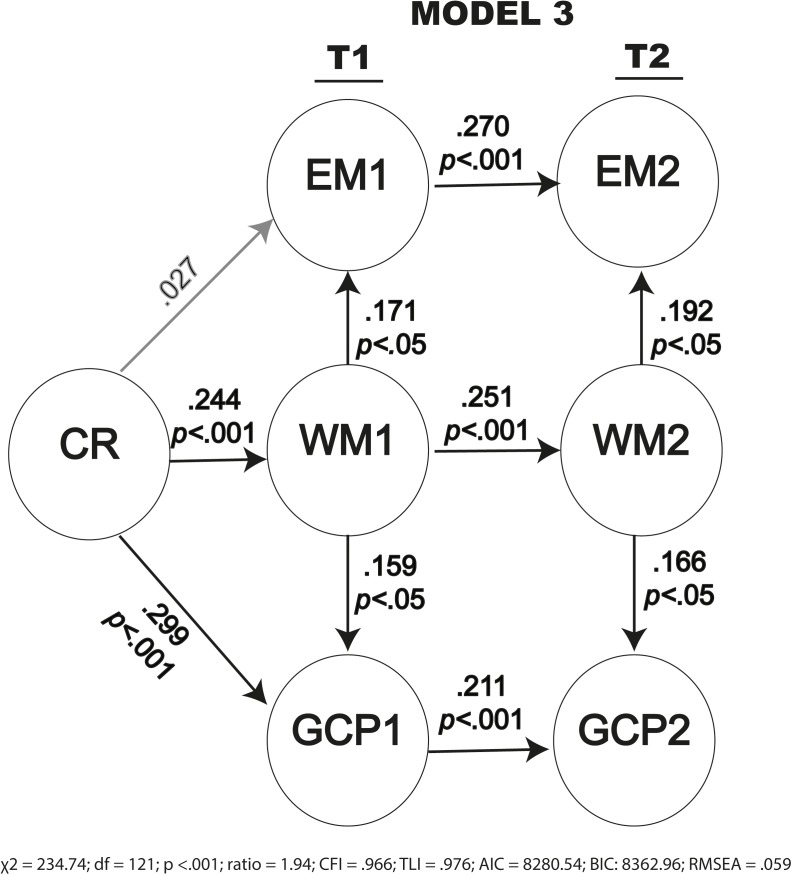
Path diagram for Model 3 with the structural parameter estimates.

The data on fitting and parameter estimation in **Table 3** therefore clearly establish Model 3 as the best model. This model is shown in **Figure 5**, in which the parameters are specified.

In summary, all models showed significant relationships between CR and cognitive performance, measured at baseline and at follow-up. Models 3 and 4 show that CR influences other cognitive constructs over time via working memory, with Model 3 providing the best fit to the data.

## Discussion

This follow-up study provides novel information about the impact of CR on cognitive performance over time in people with SCCs. First of all, our results confirm the robustness of the previous model of relationships between CR and cognitive performance (Lojo-Seoane et al., 2014a) and its structural stability at follow-up. This model enables reflective analysis of the temporal relationships between CR, as a construct composed of two latent variables (educational level and lifestyle), and three cognitive domains (GCP, episodic memory, and working memory) in people with SCCs. We suggest that this model may be useful for predicting the changes in cognitive performance of people with SCCs, taking into account that SCCs indicate a very early stage of MCI and dementia.

Considering the invariance of the measurement models presented, we defined the different models and analyzed the relationships between cognitive constructs. All of the models tested showed significant positive relationships between cognitive performance, measured at baseline and at follow-up, indicating that the performance at baseline is related to performance at follow-up. Models 3 and 4 clarified the effect of CR on cognitive performance via WM over time (Constantinidou et al., 2014; Lojo-Seoane et al., 2014a; Sumowski et al., 2014). Model 3 provided the best fit to the data, with favorable values for χ^2^ and degrees of freedom, adequate values of CFI and TLI and estimated RMSEA, and low values of AIC and BIC. The effects of cognitive performance constructs at baseline on the same constructs at follow-up were significant in all models when CR was included. This shows an effect of CR on cognitive performance at follow-up that is measured indirectly through cognitive performance constructs at baseline and confirms hypothesis 2, which predicted significant and positive effects of CR on cognitive performance at follow-up.

The nested models specifically showed an effect of CR on GCP and WM at follow-up (GCP2 and WM2) via the same cognitive constructs as at baseline. These results are consistent with the findings reported in previous studies (Ghisletta et al., 2006; Van Oijen et al., 2007; Lojo-Seoane et al., 2012, 2014a,b; Mitchell et al., 2012; Giogkaraki et al., 2013; Facal et al., 2014), showing that CR can influence cognitive performance over time. However, our findings differ from those reported by Vaughan et al. (2014), who found that CR influenced cognitive performance at baseline but not after 2–3 years. Although the study by Vaughan and colleagues and our current study coincide regarding the invariance of structures of the corresponding measurement models, the discrepancy may be explained by taking into account that the measurement model used by the aforementioned authors to form the CR construct only included indicators related to lifestyle (such as reading books, playing games, and computer activities). The current model represents a more complete view of CR by integrating two latent variables: lifestyle, including indicators of cultural and social activities, and education, formed by indicators of years of education, occupational attainment, vocabulary knowledge and reading habits. Furthermore, Vaughan et al. (2014) used the digits backward test to estimate WM, while we used a more complex measure of WM, which may be a better estimate of the construct. Another difference between the two studies is the method used to analyze the influence of CR over time. We examined the influence of CR on cognitive performance by considering raw scores at baseline and at follow-up, whereas Vaughan et al. (2014) analyzed the influence of CR on the difference between scores at T2 and at T1.

Different methods of measuring CR are described in the relevant literature. While some authors use more measures related to the educational level such as vocabulary, years of schooling and reading ability (Giogkaraki et al., 2013; Facal et al., 2014), others use CR proxies related to lifestyle and leisure activities (Ghisletta et al., 2006; Vaughan et al., 2014). In this respect, the novelty of our contribution is the inclusion of numerous indicators related to two latent variables, i.e., educational level and lifestyle, which form the CR construct (Lojo-Seoane et al., 2014a).

We analyzed the effects of WM on the other cognitive constructs at baseline and follow-up in Models 2, 3, and 4. The three models showed indirect effects of the CR cognitive constructs at baseline through the WM at baseline. Model 2 showed significant effects of WM at baseline on other cognitive constructs at follow-up and the best fit Model 3, and even Model 4, confirmed significant indirect effect of CR via WM at follow-up on episodic and GCP at follow-up. These findings confirm that CR also influences cognitive performance at follow-up through WM (hypothesis 3) and that episodic memory and GCPs are closely related to WM performance. These findings are consistent with previous findings emphasizing the important and discriminating involvement of WM in most cognitive assessment tasks (Engle, 2002; Conway et al., 2003; Gagnon and Belleville, 2011). Gagnon and Belleville (2011) studied the role of WM span across the retention interval, showing that the effect of retention interval on the amplitude of WM was a good measure for discriminating between healthy participants and people with MCI or AD. Verhaeghen (2011) also reported a series of meta-analyses on aging and executive control, concluding that WM is one of cognitive functions that best explain age-related differences in the performance of many cognitive tasks. Evidence for the mediating role of WM in the relationship of CR on other cognitive constructs or functions has been obtained in studies involving different types of neurological damage/disorders such as traumatic brain injury (Sandry et al., 2015), multiple sclerosis (Sandry and Sumowski, 2014) and MCI (Constantinidou et al., 2014). Sandry and Sumowski (2014) suggested WM capacity as a possible means whereby intellectual enrichment, as a proxy for CR, helps to preserve long-term memory in participants with multiple sclerosis. In a later study, Sandry et al. (2015) also found that WM capacity mediated the relationship between CR and long-term memory in participants with traumatic brain injury. In the same way, Constantinidou et al. (2014) reported that the effect of age on verbal episodic memory was mediated by WM capacity and observed that years of education influenced the rate of age-related decline in immediate verbal episodic memory. These findings help us to understand the relationship between CR and cognitive performance, considering WM as a mechanism by which CR may exert its protective effect on other cognitive domains. We speculate that CR will have a protective effect on WM, because education and lifestyle life contribute to the development of strategies throughout life that may improve the capacity to maintain and simultaneously manipulate information. We suggest that CR mainly protects functions involving greater availability of processing resources from WM. Regarding this suggestion, we hypothesize that the benefit of CR will be reduced when WM is impaired. However, further research on the effects of CR on cognitive performance is required in order to consider the deterioration in WM and to explain some findings indicating that CR is not associated with performance in some domains (Arcara et al., 2017).

## Conclusion

Our findings confirm that CR model proposed in the previous study is useful for measuring the influence of CR on cognitive performance, specifically on Episodic Memory, Working Memory, and GCP, in a sample of adults with subjective cognitive complaints evaluated at baseline and at a follow-up after an interval of about 18 months. The findings also highlight the positive effect of CR on cognitive performance at baseline and at follow-up and confirm the mediating role of working memory on episodic memory and GCP at each of the evaluation times in people with subjective cognitive complaints, who may be at risk of suffering mild cognitive impairment. The mediating effect of working memory highlights that education, occupational attainment, reading habits and vocabulary, and participation in social and cultural activities, all of which are components of CR, may contribute to enhancing mechanisms that enable maintenance and simultaneous manipulation of information and favor performance of tasks involving other cognitive constructs at each of the evaluation times. The findings suggest the importance of participating in activities that help improve CR and taking into account CR in cognitive assessments. Likewise evaluating and training working memory in older adults with subjective cognitive complaints is important because the direct and indirect effects of CR on episodic memory and GCP may influence the possible progress from normal aging to MCI and dementia (Richter et al., 2015; Saunders et al., 2015).

This study has some limitations, because it includes only one follow-up evaluation and considers a whole sample without distinguishing between individuals with mild cognitive impairment and cognitively healthy individuals. In future studies, we will test this structural model over a longer period, taking into account a third evaluation which is being completed in our current longitudinal project. Further research is needed to explore the effect of CR on the progress of different subtypes of individuals with subjective cognitive complaints, such as those diagnosed with MCI and those confirmed as healthy controls, taking into account the possible transition between diagnostic states and possible progression to dementia (Facal et al., 2015). Although we have shown that there were no differences in two important CR proxies and only slight differences in measures of episodic memory between participants who attended and those who did not attend the follow-up, further longitudinal research is required to study attrition and to enable general conclusions about the effects of CR to be reached.

## Author Contributions

CL-S, DF, AP, and OJ-R designed the study, conducted the research, analyzed the data, contributed to writing the manuscript, and revised and approved the final version. JG-O conducted the research, analyzed the data, contributed to writing the manuscript and revised and approved the final version.

## Conflict of Interest Statement

The authors declare that the research was conducted in the absence of any commercial or financial relationships that could be construed as a potential conflict of interest.
